# Changes in resistance among coliform bacteraemia associated with a primary care antimicrobial stewardship intervention: A population-based interrupted time series study

**DOI:** 10.1371/journal.pmed.1002825

**Published:** 2019-06-07

**Authors:** Virginia Hernandez-Santiago, Peter G. Davey, Dilip Nathwani, Charis A. Marwick, Bruce Guthrie

**Affiliations:** 1 Division of Population and Behavioural Sciences, School of Medicine, University of St Andrews, St Andrews, United Kingdom; 2 Division of Population Health and Genomics, School of Medicine, University of Dundee, Dundee, United Kingdom; 3 Academic Health Sciences Partnership in Tayside, Ninewells Hospital and Medical School, Dundee, United Kingdom; 4 Usher Institute of Population Health Sciences and Informatics, College of Medicine and Veterinary Medicine, University of Edinburgh, Edinburgh, United Kingdom; University of Bern, SWITZERLAND

## Abstract

**Background:**

Primary care antimicrobial stewardship interventions can improve antimicrobial prescribing, but there is less evidence that they reduce rates of resistant infection. This study examined changes in broad-spectrum antimicrobial prescribing in the community and resistance in people admitted to hospital with community-associated coliform bacteraemia associated with a primary care stewardship intervention.

**Methods and findings:**

Segmented regression analysis of data on all patients registered with a general practitioner in the National Health Service (NHS) Tayside region in the east of Scotland, UK, from 1 January 2005 to 31 December 2015 was performed, examining associations between a primary care antimicrobial stewardship intervention in 2009 and primary care prescribing of fluoroquinolones, cephalosporins, and co-amoxiclav and resistance to the same three antimicrobials/classes among community-associated coliform bacteraemia. Prescribing outcomes were the rate per 1,000 population prescribed each antimicrobial/class per quarter. Resistance outcomes were proportion of community-associated (first 2 days of hospital admission) coliform (*Escherichia coli*, *Proteus spp*., or *Klebsiella spp*.) bacteraemia among adult (18+ years) patients resistant to each antimicrobial/class. 11.4% of 3,442,205 oral antimicrobial prescriptions dispensed in primary care over the study period were for targeted antimicrobials. There were large, statistically significant reductions in prescribing at 1 year postintervention that were larger by 3 years postintervention when the relative reduction was −68.8% (95% CI −76.3 to −62.1) and the absolute reduction −6.3 (−7.6 to −5.2) people exposed per 1,000 population per quarter for fluoroquinolones; relative −74.0% (−80.3 to −67.9) and absolute reduction −6.1 (−7.2 to −5.2) for cephalosporins; and relative −62.3% (−66.9 to −58.1) and absolute reduction −6.8 (−7.7 to −6.0) for co-amoxiclav, all compared to their prior trends. There were 2,143 eligible bacteraemia episodes involving 2,004 patients over the study period (mean age 73.7 [SD 14.8] years; 51.4% women). There was no increase in community-associated coliform bacteraemia admissions associated with reduced community broad-spectrum antimicrobial use. Resistance to targeted antimicrobials reduced by 3.5 years postintervention compared to prior trends, but this was not statistically significant for co-amoxiclav. Relative and absolute changes were −34.7% (95% CI −52.3 to −10.6) and −63.5 (−131.8 to −12.8) resistant bacteraemia per 1,000 bacteraemia per quarter for fluoroquinolones; −48.3% (−62.7 to −32.3) and −153.1 (−255.7 to −77.0) for cephalosporins; and −17.8% (−47.1 to 20.8) and −63.6 (−206.4 to 42.4) for co-amoxiclav, respectively. Overall, there was reversal of a previously rising rate of fluoroquinolone resistance and flattening of previously rising rates of cephalosporin and co-amoxiclav resistance. The limitations of this study include that associations are not definitive evidence of causation and that potential effects of underlying secular trends in the postintervention period and/or of other interventions occurring simultaneously cannot be definitively excluded.

**Conclusions:**

In this population-based study in Scotland, compared to prior trends, there were very large reductions in community broad-spectrum antimicrobial use associated with the stewardship intervention. In contrast, changes in resistance among coliform bacteraemia were more modest. Prevention of resistance through judicious use of new antimicrobials may be more effective than trying to reverse resistance that has become established.

## Abstract 2

Charis Marwick and colleagues report on changes in antimicrobial use and bacterial resistance associated with a stewardship intervention in Scotland.

## Author summary

**Why was this study done?:**

- Antimicrobial resistance is an increasing global public health threat, and antimicrobial use in humans is a key driver of resistance development. The majority of antimicrobial prescribing occurs in the community, and there is evidence that a significant proportion of such prescribing is unnecessary.
- Antimicrobial stewardship interventions aim to improve antimicrobial use. There is increasing evidence that they are effective in influencing rates of the targeted prescribing in both primary and secondary care, but there is much less reliable evidence on their effect on resistance.
- A primary care antimicrobial stewardship intervention in the Tayside region of Scotland, UK, in 2009 was intended to reduce broad-spectrum antimicrobial use in the community. We used this opportunity to examine changes associated with the intervention in prescribing of three individual targeted antimicrobials and changes in resistance to these antimicrobials among community-associated bloodstream infections using routinely collated population data.

**What did the researchers do and find?:**

- We conducted segmented regression analyses of interrupted time series data on community prescribing of fluoroquinolones, cephalosporins, and co-amoxiclav between 2005 and 2012 and on resistance to the same antimicrobials among common gram-negative bloodstream infections (*E*. *coli*, *Klebsiella spp*., and *Proteus spp*.) identified on admission to hospital. We modelled immediate changes in rates and in trends associated with the stewardship intervention and estimated changes in prescribing (compared to predicted rates if the intervention had not occurred) 1 and 3 years later, and in resistance 1.5 and 3.5 years later (incorporating a prespecified expected 6-month delay between changes in prescribing and changes in resistance).
- We found large and sustained reductions in prescribing of all three antimicrobials associated with implementation of the stewardship intervention.
- We found reductions in coliform bacteraemia resistance rates associated with the intervention that were significant for fluoroquinolones and cephalosporins by 3.5 years but not for co-amoxiclav. The reductions in resistance took much longer than reductions in prescribing to become evident, and relative reductions were more modest. The overall pattern was of flattening rather than reversal of previously rising resistance rates.

**What do these findings mean?:**

- Antimicrobial stewardship interventions in primary care have a role in containing antimicrobial resistance in serious infections.
- Very large changes in prescribing were associated with much smaller changes in resistance. Preventing development of resistance through careful use of new antimicrobials may be more effective than attempting to reverse established resistance.

## Introduction

Increasing antimicrobial resistance (AMR) is a major global public health threat [1,2]. An estimated 2 million people have resistant infections in the United States annually, with associated longer hospital stays and more costly antimicrobial treatment; an estimated 25,000 associated deaths occur annually in the European Union, and 23,000 in the US [3,4]. Coliforms, principally *E*. *coli*, are the commonest cause of bacteraemia, with incidence rates rising internationally [5,6]. Resistant *E*. *coli* bacteraemia is associated with higher mortality and poorer health outcomes, at least partly due to increased risk of initial antimicrobial therapy being inappropriate [7–11].

Higher antimicrobial use is associated with increased resistance at both population [12] and individual [13] levels. Up to one-third of ambulatory care antimicrobial use is estimated to be inappropriate [14], with widespread inappropriate use of broad-spectrum antimicrobials [15]. Antimicrobial use is therefore considered the most modifiable cause of AMR [12,16]. Antimicrobial stewardship interventions have been widely implemented [17], with demonstrated reductions in targeted prescribing, but the effect on AMR is as yet unclear because few studies of antimicrobial stewardship interventions robustly examine the effects on resistance [18].

The most recent systematic review and meta-analysis of the effect of stewardship on subsequent AMR included 32 studies and reported that stewardship interventions in hospitals were associated with reductions in colonisation and infection with multi-drug–resistant gram-negative bacteria [19]. However, only two (6%) of the included studies were judged to be high-quality by the review authors, not least because almost all included studies had an uncontrolled before–after design, which is unreliable (particularly in this context, in which interventions are often triggered by outbreaks, which usually remit without any intervention, and include changes to infection control as well as prescribing). In the most recent systematic reviews of antimicrobial stewardship interventions selecting for higher-quality studies, effects on AMR were examined by only 15 (7%) included studies of hospital-based stewardship interventions, and only four (10%) included studies of community-based interventions [18,20]. All four community-based studies examined *Streptococcus pneumoniae* resistance, with only one showing declining macrolide resistance following reductions in community macrolide use [21]. Although restricting the use of a new antimicrobial is expected to minimise the development of resistance, it therefore remains uncertain whether reductions in existing antimicrobial use will translate into lower resistance, particularly in the community, where most antimicrobials are prescribed [21,22].

A primary care stewardship intervention implemented across the National Health Service (NHS) Tayside region of Scotland, UK, in 2009 was associated with large (>50%) reductions in total aggregate primary care prescribing of four broad-spectrum antimicrobials in primary care (fluoroquinolones, cephalosporins, co-amoxiclav, and clindamycin; targeted primarily because all are associated with *Clostridium difficile* infection) [23]. In the years since the intervention, serious gram-negative (particularly coliform) infection and antimicrobial resistance have been recognised as increasingly important problems, with three of the antimicrobials targeted in the intervention commonly used in treatment of coliform infections (coliform bacteria are intrinsically resistant to clindamycin). The aim of this study was to separately examine changes in prescribing of fluoroquinolones, cephalosporins, and co-amoxiclav over a longer period and to examine whether there were any associated changes in resistance to these antimicrobials in patients admitted to hospital with community-associated coliform bacteraemia.

## Methods

### Study context and antimicrobial stewardship intervention

In the Tayside region of Scotland, all healthcare is provided by the NHS with the exception of some less complicated elective surgery. All residents are registered with a single general practice providing primary medical care, and the regional NHS microbiology laboratory carries out all culture and sensitivity testing. Primarily in response to concerns about rising rates of *C*. *difficile* infection, in 2009, NHS Tayside implemented a multifaceted antimicrobial stewardship intervention to reduce the use of broad-spectrum antimicrobials in primary care. The intervention consisted of prescriber education, guideline development and dissemination, change of the primary care antimicrobial guideline format and content, feedback of comparative prescribing data at practice level with target setting in relation to formulary adherence, and small financial incentives to participate in improvement work (approximately £390/€441/US$497 for an average-sized practice with approximately 5,500 registered patients).

The educational component of the intervention comprised both written guidance and pharmacist-facilitated educational outreach (academic detailing) to practices. Written educational material included revised antimicrobial guidance and a special edition of the Tayside Prescriber newsletter focused on raising awareness about *C*. *difficile* infection, including avoiding broad-spectrum antimicrobials associated with it. Educational outreach included face-to-face dissemination of the new antimicrobial prescribing guidance to practices and general practitioners and discussion of the prescribing targets set for primary care. In parallel, practices’ antimicrobial prescribing was audited at both practice and individual prescriber level, with feedback of prescribing rates compared to other practices in the region. Feedback data informed target setting for each practice with the aim of increasing formulary adherence, involving practices agreeing to reduce fluoroquinolone use in general and for respiratory infections in particular and to reduce antimicrobial use without a documented indication. Practices also agreed to implement a practice policy for dealing with inappropriate or telephone requests for antimicrobials.

We have previously reported that the intervention was associated with a rapid, large, and sustained decrease in an aggregate measure of total targeted broad-spectrum antimicrobial prescribing (fluoroquinolones, cephalosporins, co-amoxiclav, and clindamycin) in all age groups [23].

### Data

The University of Dundee Health Informatics Centre (HIC) provided data from 1 January 2005 to 31 December 2015 on all blood culture isolates in the region (total including negative results = 157,229, total gram-negative bacteraemia = 4,701), community-dispensed antimicrobials (total 3,442,205 prescriptions), patient demography (mean population per study calendar quarter = 424,014), hospital admissions, and mortality. Linkage between data sets at individual patient level was done using the NHS Scotland unique patient identifier (the Community Health Index [CHI] number). The CHI number is used to identify patients during all NHS Scotland healthcare episodes and is consistent across all data sets. Details of the data sets and the cleaning and linkage processes are provided as supporting information (S1 Text).

### Protocol, ethics, and reporting

This observational study was conducted as part of VHS’s doctoral research and did not have a published protocol or signed, dated analysis plan. However, all the primary analyses reported in the main paper were specified prior to examining the outcome data, with the sensitivity analyses designed post hoc informed by examination of the time trends on request of the statistical reviewer.

HIC Standard Operational Procedures (SOPs) have been approved by both the NHS East of Scotland Research Ethics Committee and the relevant Caldicott Guardians (who have legal responsibility for approving use of unconsented NHS patient data). Ethics committee review of individual studies is not required provided that HIC SOPs are followed, including that all data analysis is carried out using anonymised data held in the ISO27001 and NHS Scotland accredited HIC Safe Haven.

The study is reported in accordance with the REporting of studies Conducted using Observational Routinely collected health Data (RECORD) statement (S1 RECORD Checklist) [24].

### Study population

The population for the prescribing analysis included the whole population registered with a general practitioner in NHS Tayside from 1 January 2005 to 31 March 2012. For the resistance analysis, patients were included if they were aged 18+ years and had a hospital admission with community-associated coliform bacteraemia from 1 January 2005 to 31 December 2015. Coliform bacteraemia was defined as isolation of any of *E*. *coli*, *Klebsiella spp*., or *Proteus spp*. in a blood culture specimen. Community-associated was defined as an isolate from a blood culture taken on days 0, 1, or 2 of a hospital admission in patients with no previous hospital admission in the 30 days prior to index admission date. Only the first eligible isolate per patient per calendar quarter was included.

### Outcomes

We examined changes in prescribing for each of the three targeted broad-spectrum antimicrobials that are used in gram-negative infection (fluoroquinolones, cephalopsorins, and co-amoxiclav), measured as the rate of patients per 1,000 population exposed to each antimicrobial in primary care in each calendar quarter. We then examined changes in resistance to these three antimicrobials/classes in community-associated coliform bacteraemia isolates, measured as the quarterly rate of resistance per 1,000 eligible isolates. Resistance was defined as in vitro resistance reported by the Tayside Microbiology laboratory, which used Vitek systems (Vitek until 2007, then Vitek 2 subsequently) and Clinical and Laboratory Standards Institute (CLSI)-recommended minimum inhibitory concentrations (MICs) during the study period. We classified isolates in which the MIC indicated intermediate susceptibility as resistant to that antimicrobial. Prescribing outcomes were measured quarterly from the first quarter of 2005 to the first quarter of 2012, inclusive, and resistance outcomes were measured quarterly from the first quarter of 2005 to the fourth quarter of 2015, inclusive.

### Statistical analysis

Segmented regression analysis of interrupted time series data plotted quarterly was used to examine changes in both prescribing and resistance associated with implementation of the antimicrobial stewardship intervention. Time series analysis is generally acknowledged to be a robust quasiexperimental method for analysing the effect of interventions that have not been or cannot be randomised. It allows the statistical assessment of how an intervention is associated with change in an outcome of interest both immediately and over time [25]. A time series is repeated observations (aggregate measures at each time point, for example, proportions or means) of a particular outcome over time, which is then divided (interrupted) into time periods before and after an intervention. Segmented regression analysis evaluates both changes in level (i.e., any step change) immediately after the intervention and changes in slope in the postintervention period compared to the preintervention period, which serves as the control. Segmented regression controls for the effect of secular time trends, avoiding bias commonly present in uncontrolled before-and-after comparison of mean rates.

The stewardship intervention was implemented at the start of the second quarter of 2009, and for the prescribing analysis, this was chosen to be the first time point after the intervention, giving 17 time points before the intervention and 12 time points in the postintervention segment, as prescribing data from 2005 to 2012 were included in the model. Constraining the postintervention time period in this way reduced the risks that assumptions of linearity will be violated and that other stewardship interventions will influence the outcome but includes sufficient time points to evaluate intervention effects, including their sustainability. For resistance analysis purposes, we a priori assumed that there would be a 6-month lag between intervention implementation and any associated change in resistance, based on evidence that individual exposure to antimicrobials is associated with subsequent increased resistance to those antimicrobials for up to 6 months [26–29]. The segmented regression model for resistance therefore assumed an interruption at the fourth quarter of 2009 (rather than the second quarter) and included data to the end of 2015, giving 19 preinterruption and 25 postinterruption time points. Models estimated the baseline level and trend in prescribing and resistance rates before the interruption and any step change or change in trend following the interruption.

We fitted an autoregressive model to analyse the changes in both prescribing and resistance. The core model fitted was as follows [25]:

$$Y_{t}=\beta_{0}+\beta_{1}\;*\;time_{t}+\beta_{2}\;*\;interruption+\beta_{3}\;*\;time\_after\_interruption_{t}+e_{t},$$

where Y_t_ is the outcome (for example, the rate per 1,000 coliform bacteraemia with resistance to a specified antimicrobial) at time t; time is a continuous variable, indicating the number of quarters from the start of the study period; interruption is an indicator for time points before and after the interruption; and time after interruption is a continuous variable, indicating the number of quarters after the interruption. β_0_ estimates the baseline level of the outcome at the start of the study, β_1_ estimates the baseline trend before the interruption, β_2_ estimates the immediate change in level of the outcome after the interruption, and β_3_ estimates the change in the trend after the interruption.

For prescribing outcomes, we estimated absolute and relative changes at 1 and 3 years after the intervention. For resistance, since the model interruption was a priori defined to be 6 months after the start of the antimicrobial stewardship intervention, results are reported as absolute and relative change at 1.5 and 3.5 years after the stewardship intervention (a 6-month lag, then 1 and 3 years’ follow-up from the modelled interruption). The absolute change was calculated as the difference between model estimates of the predicted outcome if prior trends had continued and the outcome given any intervention effect. The relative change is the absolute change as a proportion of the outcome rate that the model predicted would have occurred if prior trends had continued. We used the bootstrapping method proposed by Zhang [30], implementing an adaptation of Zhang’s published SAS macro to calculate the 95% CIs for both absolute and relative percentage change estimates.

For all models, we examined whether there was significant autocorrelation up to fourth-order autocorrelation using the Durbin–Watson statistic and visual inspection of autocorrelation and partial autocorrelation plots. Lag terms were fitted as required to adjust for significant autocorrelation and were included in the models if they were statistically significant and improved model fit. In a similar way, seasonality was explored and adjusted for in the prescribing models as required. Model outputs for lag and seasonal terms have no meaningful interpretation in terms of estimating changes associated with the intervention, so they are not reported.

**Fig 1:**
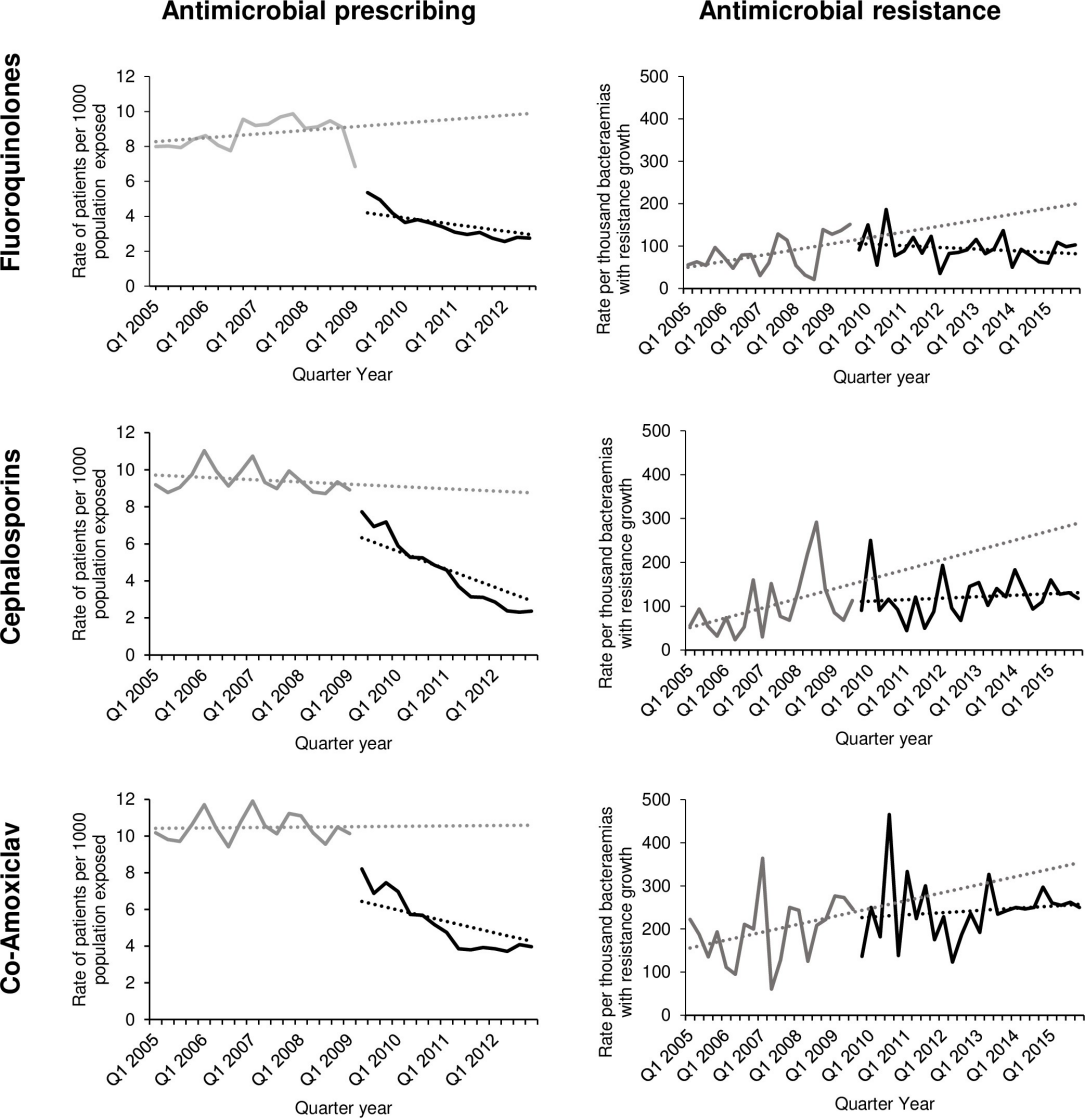
Rates of community antimicrobial exposure 2005 to 2012 and rates of resistance in community-associated coliform bacteraemia episodes 2005 to 2015 in Tayside in relation to a primary care antimicrobial stewardship intervention implemented in the second calendar quarter of 2009.

Because of the shape of the resistance time series (Fig 1, right-hand panels), we conducted two types of post hoc sensitivity analysis for resistance to each of the three antimicrobials. First, we modelled the outcome against time without fitting any interruption and compared model fit using the Akaike Information Criterion (AIC) [31] with the model including the interruption for each antimicrobial. Next, we excluded the first year’s data from each resistance time series and refitted the models using data from 1 January 2006 to 31 December 2015 and compared model outputs and estimated absolute and relative changes with the results of the prespecified analyses.

## Results

A total of 3,442,205 oral antimicrobial prescriptions were dispensed in primary care in Tayside over the study period. Amoxicillin was by far the most prescribed antimicrobial, representing 30.1% of all antimicrobial prescriptions, followed by tetracyclines (11.8%), trimethoprim (11.4%), flucloxacillin (10.1%), and macrolides (9.5%). In total, the three targeted broad-spectrum antimicrobials included here accounted for 11.2% of all antimicrobial prescribing.

There were 2,143 eligible bacteraemia episodes from 2,004 patients over the whole period examined (mean age 73.7 [SD 14.8] years; 51.4% women), increasing from 136 in 2005 to 234 in 2015 (Table 1). There was a significant upwards trend in eligible bacteraemia episodes (increasing by 0.2 per 100,000 population per quarter, *p* = 0.01) prior to the intervention but no significant change in level or trend associated with the intervention (*p* = 0.26 and *p* = 0.53, respectively) (S1 Fig and S1 Table). Over the whole period, *E*. *coli* was isolated in 81.8% of eligible bacteraemia episodes, compared to *Klebsiella spp*. in 12.0% and *Proteus spp*. in 6.2%, with little change in these proportions over time. Mean length of stay fell slightly, and 30-day mortality did not change during the study period (Table 1). Across the entire time period, mean resistance rates were 9.0% for fluoroquinolones, 10.6% for cephalosporins, and 22.6% for co-amoxiclav. The proportions resistant to more than one of the three antimicrobials were low, with a maximum 12.8% (24 of 188) resistant to any two and 4.9% (10 of 203) resistant to all three in any study year, in 2009 and 2010, respectively (S2 Table).

**Table 1:** CB episodes per study year in total, by study eligibility, by organism, and by length of stay and mortality for patients with eligible bacteraemia in each study year.

Year	Total number of CB isolates^a^	Number of patients with CB on day 0, 1, or 2 of a hospital admission^b^	Number of eligible CA-CB^c^	Number of *E*. *coli* CA-CB (% of CA-CB)	Number of *Klebsiella* CA-CB (% of CA-CB)	Number of *Proteus* CA-CB (% of CA-CB)	Median length of stay CA-CB^d^ in days (IQR)	Number of CA-CB patients died within 30 days^e^ (% of CA-CB)
		% (95% CI) of all CB	% (95% CI) of all CB					
2005	313	190	136	108 (79.4)	17 (12.5)	11 (8.1)	12.8 (11.7 to 14.4)	28 (20.6)
		60.7% (55.0 to 66.1)	43.4% (37.9 to 49.1)					
2006	293	194	132	100 (75.8)	22 (16.7)	10 (7.6)	16.2 (16.0 to 16.6)	23 (18.6)
		66.2% (60.4 to 71.5)	45.1% (39.2 to 50.9)					
2007	372	219	149	115 (77.1)	19 (12.7)	15 (10.1)	15.9 (14.1 to 19.2)	23 (15.7)
		58.9% (53.7 to 73.9)	40.1% (35.1 to 45.2)					
2008	388	235	153	128 (83.7)	14 (9.1)	11 (7.2)	18.1 (16.1 to 22.5)	13 (8.1)
		60.6% (55.5 to 65.4)	39.4% (34.6 to 44.5)					
2009	490	278	188	158 (84.0)	20 (10.6)	10 (5.3)	17.1 (14.7 to 19.7)	28 (14.9)
		56.7% (52.1 to 61.1)	38.4% (34.1 to 42.8)					
2010	479	298	203	174 (85.7)	22 (10.8)	7 (3.4)	13.6 (12.1 to 14.6)	37 (18.4)
		62.2% (57.7 to 66.5)	42.4% (37.9 to 46.9)					
2011	501	323	220	174 (79.1)	35 (15.9)	11 (5.0)	15.9 (13.4 to 18.2)	20 (8.5)
		64.5% (60.1 to 68.6)	43.9% (39.5 to 48.4)					
2012	509	342	244	193 (79.1)	33 (13.5)	18 (7.4)	16.8 (13.6 to 19.4)	40 (16.3)
		67.2% (62.9 to 71.2)	47.9% (43.5 to 52.3)					
2013	491	328	231	188 (81.4)	30 (13.0)	13 (5.6)	12.9 (12.3 to 13.2)	38 (16.3)
		66.8% (62.4 to 70.9)	47.1% (42.6 to 51.6)					
2014	480	352	253	208 (82.2)	34 (13.4)	11 (4.3)	12.4 (11.2 to 14.0)	39 (15.4)
		73.3% 9(67.1 to 79.2)	52.1% (47.1 to 57.2)					
2015	486	328	234	196 (83.8)	22 (9.4)	16 (6.8)	12.1 (11.4 to 13.4)	28 (11.9)
		67.5% (63.1 to 71.6)	48.1% (43.6 to 52.7)					

^a^CB defined as *E*. *coli*, *Klebsiella spp*., or *Proteus spp*. isolated from blood culture; numbers include isolates from any point in admission and multiple isolates from the same patient during an admission or in multiple admissions.

^b^Patients with one or more CB isolates on day 0, 1, or 2 of hospital admission; numbers include multiple admissions per patient per year.

^c^CA-CB case is defined as CB on day 0, 1, or 2 of admission in a patient who has not been in hospital in the 30 days before the admission and without a previous CA-CB in the same calendar quarter.

^d^Length of stay is median length of hospital stay (and IQR) in days of the admission during which the CA-CB blood culture was taken.

^e^30-day mortality includes all deaths within 30 days of admission, including out of hospital mortality.

**Abbreviations:** CA-CB, community-associated CB; CB, coliform bacteraemia.

### Changes in prescribing

Table 2 shows the results of the interrupted time series analysis, and Table 3 reports the estimated changes associated with the intervention. There was significant autocorrelation in the prescribing time series for co-amoxiclav but not for fluoroquinolones or cephalosporins, according to the Durbin–Watson statistic and examination of autocorrelation plots. Adding a seasonal (in which calendar quarters 1 and 4 are winter and 2 and 3 are summer) term to the co-amoxiclav model improved model fit and accounted for autocorrelation (S3 Table). Consistent with our previous work examining an aggregate measure of broad-spectrum antimicrobial prescribing [23], there were large decreases in individual targeted antimicrobials used in gram-negative infection: fluoroquinolones, cephalosporins, and co-amoxiclav (Fig 1, left-hand panel). There were no significant trends prior to the intervention for any of these antimicrobials. However, following the intervention, there were statistically significant immediate reductions in prescribing of −3.8 (95% CI −4.7 to −3.0, *p* < 0.001) per 1,000 population dispensed per quarter for fluoroquinolones, of −1.2 (−1.9 to −0.5, *p* = 0.001) for cephalosporins, and of −1.9 (−2.6 to −1.3, *p* < 0.001) for co-amoxiclav (Table 2). The intervention was also associated with significant changes in trend following the intervention with further significant, additional decreases of −0.2 (95% CI −0.4 to −0.1, *p* < 0.001), −0.4 (95% CI −0.5 to −0.3, *p* < 0.001), and −0.4 (95% CI −0.5 to −0.3, *p* < 0.001) patients per 1,000 population dispensed per quarter for fluoroquinolones, cephalosporins, and co-amoxiclav, respectively. Given preintervention trends, fluoroquinolone prescribing (which was the main target of the intervention) at 1 and 3 years postintervention was estimated to be −53.4% (95% CI −58.9% to −47.7%) and −68.8% (−76.3% to −62.1%) lower than expected. These equate to absolute reductions of −4.7 (−5.5 to −4.0) and −6.3 (−7.6 to −5.2) people exposed per 1,000 population per quarter at 1 and 3 years postintervention, respectively. There were also large and statistically significant relative reductions in both cephalosporin and co-amoxiclav prescribing at 1 and 3 years postintervention (Table 3).

**Table 2:** Results of interrupted time series analysis of changes in prescribing of targeted antimicrobials associated with the stewardship intervention.

	Baseline prescribing (rate per 1,000 population at start of time series)^a^	Baseline trend (increase [+] or decrease [−] per quarter of rate per 1,000 population)^a^	Step change postintervention (increase [+] or decrease [−] in rate per 1,000 population)^a^	Change in trend postintervention (increase [+] or decrease [−] in rate per quarter per 1,000 population)^a^
Fluoroquinolones	7.9 (7.2 to 8.4)	+0.04 (−0.01 to 0.1)	−3.8 (−4.7 to −3.0)	−0.2 (−0.4 to −0.1)
Cephalosporins	9.3 (8.8 to 9.8)	−0.04 (−0.09 to 0.006)	−1.2 (−1.9 to −0.5)	−0.4 (−0.5 to −0.3)
Co-amoxiclav^b^	11.1 (10.5 to 11.7)	−0.01 (-0.05 to 0.03	−1.9 (−2.6 to −1.3)	−0.4 (−0.5 to −0.3)

^a^Rate of patients per 1,000 population exposed to each antimicrobial in each quarter.

^b^A term for ‘season’ (1 for winter months, i.e., October to March, and 2 for summer months) was fitted for co-amoxiclav prescribing to account for seasonality.

**Table 3:** Estimated absolute and relative change, compared to levels predicted by prior trends, in prescribing of targeted antimicrobials at 1 and 3 years after primary care stewardship intervention.

	Absolute change at 1 year postintervention compared to predicted (patients exposed per 1,000 population)	Absolute change at 3 years postintervention compared to predicted (patients exposed per 1,000 population)	Relative change at 1 year postintervention compared to predicted	Relative change at 3 years postintervention compared to predicted
Fluoroquinolones	−4.7 (−5.5 to −4.0)	−6.3 (−7.6 to −5.2)	−53.4% (−58.9 to −47.7)	−68.8% (−76.3 to −62.1)
Cephalosporins	−2.9 (−3.5 to −2.2)	−6.1 (−7.2 to −5.2)	−33.9% (−39.2 to −28.3)	−74.0% (−80.3 to −67.9)
Co-amoxiclav	−3.6 (−4.2 to −3.1)	−6.8 (−7.7 to −6.0)	−32.7% (−36.4 to −28.9)	−62.3% (−66.9 to −58.1)

### Changes in resistance

Table 4 shows the results of the interrupted time series analysis, and Table 5 reports the estimated changes associated with the intervention. There was significant autocorrelation in the cephalosporin resistance time series but not for fluoroquinolones or co-amoxiclav. Fitting a lag 4 term improved both model fit according to AIC and the Durbin–Watson statistic, accounting for autocorrelation (S4 Table). Resistance to all three targeted antimicrobials was significantly increasing before the intervention (Fig 1, Table 4), but there were no statistically significant immediate step changes in resistance at the time of the interruption (Table 4). Preintervention, fluoroquinolone resistance was statistically significantly increasing each quarter by 3.5/1,000 (95% CI 0.5 to 6.4, *p* = 0.02) bacteraemia episodes (from a baseline rate of 46.5/1,000) with a subsequent downwards change in trend postintervention of −4.4/1,000/quarter (−7.9 to −0.9, *p* = 0.01). Cephalosporin resistance was statistically significantly increasing each quarter by 8.2/1,000 bacteraemia episodes (3.5 to 12.9, *p* = 0.001), from baseline 49.9, with a downwards change in trend postintervention of −7.0/1,000/quarter (−12.3 to −1.8, *p* = 0.03). Co-amoxiclav resistance was nonsignificantly increasing each quarter by 4.6/1,000 bacteraemia episodes (−1.0 to 10.3, *p* = 0.11) with a nonsignificant downward change in slope of −3.3/1,000/quarter (−10.4 to 3.8, *p* = 0.33).

**Table 4:** Results of interrupted time series analysis of changes in AMR to targeted antimicrobials among community-associated coliform bacteraemia associated with the stewardship intervention (modelled interruption is date of primary care antimicrobial stewardship intervention plus 6 months).

	Baseline resistance (rate per 1,000 bacteraemia episodes at start of time series)^a^	Baseline trend (increase [+] or decrease [−] per quarter of rate per 1,000 bacteraemia episodes)^b^	Step change at 6 months postintervention (increase [+] or decrease [−] in rate per 1,000 bacteraemia episodes)^b^	Change in trend at 6 months postintervention (increase [+] or decrease [−] in rate per quarter per 1,000 bacteraemia episodes)^b^
Fluoroquinolones	46.5 (13.2 to 79.8)	+3.5 (0.7 to 6.3)	−5.7 (−46.5 to 35.1)	−4.4 (−7.8 to −1.0)
Cephalosporins^c^	49.9 (4.1 to 95.6)	+8.2 (3.3 to 13.1)	−61.0 (−125.1 to 3.1)	−7.0 (−12.6 to −1.5)
Co-amoxiclav	151.5 (87.1 to 215.8)	+4.6 (−1.0 to 10.3)	−13.5 (−98.8 to 71.8)	−3.3 (−10.4 to 3.8)

^a^Rate per 1,000 community-associated coliform bacteraemia episodes resistant to each antimicrobial.

^b^Analysis prespecified an expected delay of 6 months between the stewardship intervention at the start of the second quarter of 2009 and any change in resistance. The modelled interruption is therefore the start of the fourth quarter of 2009.

^c^A lag 4 term was included for cephalosporins to account for autocorrelation.

**Abbreviations:** AMR, antimicrobial resistance.

There was a relative reduction in fluoroquinolone resistance compared to predicted by 1.5 years after the stewardship intervention (−17.2%, 95% CI −38.4 to 0.04) that increased to −34.7% (−52.3 to −10.6) by 3.5 years. These equate to absolute reductions of −27.2 (−68.8 to 7.9) and −63.5 (−131.8 to −12.8) resistant bacteraemia episodes per 1,000 per quarter by 1.5 and 3.5 years postintervention, respectively (Table 5). The overall pattern was that a rising trend in resistance was reversed (Fig 1). Statistically significant relative reductions in cephalosporin resistance of −38.2% (95% CI −56.1 to −18.8) compared to predicted were observed by 1.5 years and −48.3% (95% CI −62.7 to −32.3) by 3.5 years after the stewardship intervention. Equivalent absolute reductions were −95.0 (−157.4 to −42.4) and −153.1 (−255.7 to −77.0) resistant bacteraemia episodes per 1,000 per quarter by 1.5 and 3.5 years postintervention, respectively (Table 5). The overall effect was that a rising trend essentially flattened. Differences between observed and predicted co-amoxiclav resistance were in the direction of a reduction, consistent with flattening of a previously rising trend, but did not reach statistical significance.

**Table 5:** Estimated absolute and relative changes, compared to levels predicted by prior trends, in resistance among community-associated coliform bacteraemia at 1.5 and 3.5 years after primary care antimicrobial stewardship intervention (modelled interruption is date of primary care antimicrobial stewardship intervention plus 6 months).

	Absolute change at 1.5 years postintervention compared to predicted (rate with resistant growth per 1,000 coliform bacteraemia episodes)	Absolute change at 3.5 years postintervention compared to predicted (rate with resistant growth per 1,000 coliform bacteraemia episodes)	Relative change at 1.5 years postintervention compared to predicted (rate with resistant growth per 1,000 coliform bacteraemia episodes)	Relative change at 3.5 years postintervention compared to predicted (rate with resistant growth per 1,000 coliform bacteraemia episodes)
Fluoroquinolones	−27.2 (−68.8 to 7.9)	−63.5 (−131.8 to −12.8)	−17.2% (−38.4 to 0.04)	−34.7% (−52.3 to −10.6)
Cephalosporins	−95.0 (−157.4 to −42.4)	−153.1 (−255.7 to −77.0)	−38.2% (−56.1 to −18.8)	−48.3% (−62.7 to −32.3)
Co-amoxiclav	−34.8 (−121.8 to 38.5)	−63.6 (−206.4 to 42.4)	−11.3% (−35.9 to 18.6)	−17.8% (−47.1 to 20.8)

In the first post hoc sensitivity analyses comparing simple linear models of resistance outcomes over time to the primary models, including an interruption at 6 months after the stewardship intervention, AIC values indicated equivalent model fit (i.e., difference in AIC < 4) for all three antimicrobials (S5 Table). However, testing the specific hypothesis that the intervention was associated with changes in resistance requires fitting of an interruption, and model fit was no worse, supporting the primary analysis as reasonable, although interpretation should be cautious.

In the second post hoc sensitivity analyses comparing segmented regression resistance models that exclude 2005 data to the primary segmented regression resistance models (model outputs in S6 Table), the sensitivity analyses estimated relative changes at 1.5 and 3.5 years postintervention that for fluoroquinolones were larger but had wider 95% CI, for cephalosporins were slightly smaller with wider 95% CI, and for co-amoxiclav were slightly smaller with wider 95% CI and remained nonsignificant (S7 Table). These are in accordance with interpretation of the primary analyses, that the overall pattern is of reductions in resistance but the magnitude of the changes is modest compared to those for changes in prescribing, and not all changes are statistically significant. Again, this emphasises that interpretation of the resistance models should be cautious.

## Discussion

### Summary of main findings

In this population-based study in Scotland, UK, large reductions in community prescribing of broad-spectrum antimicrobials following an antimicrobial stewardship intervention were associated with reductions in resistance among people admitted to hospital with community-associated coliform bacteraemia. Reductions in resistance from those predicted by prior trends were statistically significant by 1.5 years for cephalosporins (where rising rates flattened) and by 3.5 years after the stewardship intervention for fluoroquinolones (where rising rates were reversed). The changes in co-amoxiclav resistance were in the direction of flattening a rising trend, although they were not statistically significant. By 3.5 years after the stewardship intervention, the absolute changes were a reduction of 63.5 isolates resistant to fluoroquinolones per 1,000 bacteraemia episodes, a reduction of 153.1 per 1,000 bacteraemia episodes resistant to cephalosporins, and a nonsignificant reduction of 63.6 per 1,000 resistant to co-amoxiclav. Multidrug resistance was rare.

### Strengths of the study

A key strength of this study is the availability of longitudinal data in a complete geographical population, allowing examination of long-term associations between a stewardship intervention and both targeted broad-spectrum antimicrobial prescribing and resistance to targeted broad-spectrum antimicrobials in serious community-associated infections. Further strengths include that the data analysed are all captured as part of routine care, meaning the likelihood of missing data is very low, and that Tayside has a very stable population, meaning observed changes are unlikely to reflect changes in the population at risk. In addition, there were no significant changes to the organisation of healthcare in Tayside over the study period, with the same two acute hospitals serving the whole population. We know that total antimicrobial prescribing in primary care did not change in association with the intervention [23,32], so it is unlikely that changes in general practitioner consultation rates or patient demand for antimicrobials influenced the findings. In addition, health-seeking behaviour is less likely to influence bacteraemia rates than positive community urine culture rates and cannot explain changes in resistance rates.

The interrupted time series analysis (ITSA) used is a robust design for evaluation of real-world interventions that cannot be randomised [33,34], and there were adequate time points before and after the intervention in our data series. In comparison with uncontrolled before–after (UBA) studies, which compare mean rates or proportions before and after an intervention, ITSA accounts for pre- and postintervention trends and changes in trend at the time of intervention. This is important for estimating intervention effects; for example, if the rate of the outcome of interest increases before an intervention and then decreases after (as happens with fluoroquinolone resistance in this analysis), then a UBA study may find that the mean outcome before and after does not change because time trends are ignored. Similarly, if the outcome was increasing preintervention and continued to increase at the same rate postintervention, a UBA study would erroneously report an increase associated with the intervention. Consistent with these limitations, UBA studies are not considered robust enough by the Cochrane Effective Practice and Organisation of Care (EPOC) Group to be included in systematic reviews, whereas ITSAs are eligible for inclusion [35].

The focus on community-associated bacteraemia is a further strength that minimises confounding from unmeasured exposure to antimicrobials in hospital. The data analysed are from one area of Scotland, but Tayside is representative (approximately 10%) of the wider Scottish population in terms of sociodemographic characteristics and not dissimilar to other regions, at least across the UK. There is no biological reason that an intervention elsewhere that is associated with similarly large changes in prescribing would not have similar associated changes in resistance, so these findings are likely to be widely generalisable.

### Limitations of the study

One weakness of the analysis is that observational studies can only examine and report association, rather than causation, and it is impossible to exclude residual confounding, which, in the case of ITSA, includes not knowing what the postintervention trends in the outcomes would have been if the intervention had not occurred. Including a control group would have strengthened the analysis, but there was no suitable control group because this intervention was implemented across the entire health board, and all other Scottish health boards implemented prescribing interventions of various kinds at around the same time in response to a national *C*. *difficile* infection outbreak. More specific to ITSA and to this data series, a weakness is the relatively low number of bacteraemia episodes per time point (median 49.5 [IQR 37.7 to 59.2]), which increases variability between time points and reduces the power to detect a true change. ITSA may also be at risk of bias if there are other major interventions in the time period examined. There were no other major changes to community prescribing or infection control policies, but there was a parallel change in the NHS Tayside hospital prescribing policy implemented at the end of 2008. The hospital policy included removing the same targeted antimicrobials/classes (as the primary care policy) from first-line indicated therapy for almost all infections. The only remaining first-line therapeutic indications in the hospital policy for targeted antimicrobials were fluoroquinolones for prostatitis, ceftriaxone for bacterial meningitis (which are both uncommon), and co-amoxiclav for severe community-acquired pneumonia (which is relatively common). It is possible that these changes had some effect on resistance among coliforms circulating in the community, even though patients with a recent hospital admission were excluded from the analysis. This is more likely for co-amoxiclav, given that its use remained higher in hospital after the policy change, and may partly explain the lack of significant reduction in co-amoxiclav resistance observed. The size and temporal relationship of the changes we observed in prescribing are consistent with these being attributable to the intervention. Given the smaller changes in resistance observed and the findings of the sensitivity analyses, we are more cautious in our interpretation of the observed changes in resistance, which reinforces the conclusion that large changes in community prescribing are associated with more modest changes in resistance at best.

### Comparison with the literature

Most antimicrobial prescribing is in the community, but the evidence base for antimicrobial stewardship in the community [20] is weaker than in the hospital [18], and few studies have robustly examined associations between community antimicrobial stewardship programs and resistance. A pilot study aiming to prospectively recruit patients admitted to hospital with urinary tract infection found an association between prior antimicrobial exposure and trimethoprim resistance in the index urine sample (crude odds ratio 3.58, 95% CI 1.18 to 10.9). The findings were limited by the small sample size, and the authors concluded that a study using routine data was required [32].

Gram-positive resistance has been shown to reduce in response to community-based stewardship. For example, pneumococcal macrolide resistance in Finland fell from 19.0% to 8.6% over 5 years following >40% reductions in macrolide use in the 1990s [21], and falling penicillin use in Iceland was associated with 25% reductions in pneumococcal penicillin resistance over 5 years [36]. Reported associations between community antimicrobial use and gram-negative resistance are less consistent. A 25% reduction in ciprofloxacin use in Israel lasting 7 months was associated with a reported reduction in fluoroquinolone resistance in urinary *E*. *coli* isolates from 12% to 9% [37]. In contrast, an 86% reduction in trimethoprim use in Sweden over 24 months was not associated with any change in resistance in urinary isolates [38], and sulphonamide resistance in the UK continued to rise in the 1990s despite the virtual ending of sulphonamide use [39].

The inconsistent relationship between antimicrobial stewardship and subsequent resistance observed in this study and others is likely related to the multiple mechanisms by which resistance arises and persists. Antimicrobial prescribing leads to selection pressures that increase resistance, but reducing antimicrobial use and therefore selection pressure will only rapidly reverse resistance if there is a significant associated fitness cost to maintaining it [40–42]. The cell wall structure in gram-positive bacteria results in higher fitness costs of resistance, consistent with observed reversibility of resistance after reductions in prescribing [21,36,43]. Fluoroquinolone resistance among *E*. *coli* has well-described associated fitness costs, but some resistance mutations confer little or no cost to bacterial fitness, and certain resistance traits may even be beneficial [42,43]. In addition, selection of resistance to a particular antimicrobial may occur in the absence of exposure to that particular antimicrobial because of mutations for resistance coexisting on the same plasmid and so being selected by other antimicrobial use [42,43]. Changes in resistance in response to stewardship interventions would therefore be predicted to have somewhat variable effects.

### Implications of the findings

The findings are consistent with primary care antimicrobial stewardship targeting broad-spectrum antimicrobial prescribing being associated with worthwhile but delayed changes in resistance in coliform bacteraemia, a serious infection. However, this is not the only potential approach to containing resistance, and it may have limited impact if there is substitution with other antimicrobials because of the risk of developing cross-resistance such as that mediated by plasmids. Alternatives include reducing total antimicrobial prescribing and short-term cycling. Reducing total antimicrobial prescribing is increasingly the strategy of choice in primary care [44], but this is complicated because of the risk of harm from undertreating high-risk groups, including the elderly, if the treatment threshold is high [45]. It will therefore be very important to collect balancing measures relating to infection outcomes in evaluating stewardship that reduces total antimicrobial use [46]. Planned, short-term cycling of recommended antimicrobials is an alternative strategy that aims to avoid sustained selection pressure of any individual antimicrobial to limit the development of resistance. There is some evidence of effectiveness of this approach in the controlled environment of intensive care units, although reported associated changes in resistance are mixed in this context [47]. Short-term cycling is limited by the small number of antimicrobials available for any indication, and its value in primary care remains to be established. Whatever strategy is chosen, the key problem remains avoiding antimicrobial prescribing when it is not necessary, and judicious use of new antimicrobials is key to reducing or containing resistance. Optimising these approaches will require improved clinical decision support and/or diagnostics at the point of care that overcome logistical, financial, and behavioural barriers to their implementation [48].

Further research is needed to better understand broader mechanisms of changes in resistance in response to antimicrobial stewardship interventions, including bacterial genomic studies in combination with population data. ITSA is an appropriate method for evaluating healthcare interventions that cannot be randomised, but the widespread application of ITSA in a timely manner requires more responsive surveillance systems that use continuous monitoring of routine data.

### Conclusion

The known mechanisms involved in gram-negative resistance are consistent with our findings that very large (>60%) reductions in community antimicrobial use can help contain resistance in coliform bacteraemia in this context but may not lower it from established rates. Notably, the associated reduction in resistance was delayed, and rising resistance only truly reversed for fluoroquinolones. Minimising broad-spectrum antimicrobial use in the community is clearly of value in containing resistance in specific serious infections, but further research is required to examine the impact on resistance more generally and other outcomes of community stewardship interventions aiming to achieve large reductions in total antimicrobial use. The findings also emphasise the critical importance of careful use of new antibiotics to minimise the initial development of resistance.

## Supporting information

S1 RECORD Checklist RECORD, REporting of studies conducted using observational routinely collected health data. (DOCX)

S1 Data Quarterly rates of prescribing and resistance. (XLSX)

S1 Text Data sets and processes for data cleaning and linkage. (DOC)

S1 Fig Admission rate (per 100,000 population) with community-associated coliform bacteraemia in Tayside 2005–2015. (TIF)

S1 Table ITSA of rate of admission with community-associated coliform bacteraemia (modelled interruption is date of primary care antimicrobial stewardship intervention plus 6 months). ITSA, interrupted time series analysis.
(DOCX)

S2 Table Rates of resistance to individual antimicrobials and combinations of targeted antimicrobials among eligible community-associated bacteraemia episodes per study year. (DOCX)

S3 Table Assessment and accounting for autocorrelation in prescribing time series with final model selection. (DOCX)

S4 Table Assessment and accounting for autocorrelation in resistance time series with final model selection. (DOCX)

S5 Table Sensitivity analysis 1: Comparing model fit for simple linear regression models of resistance outcomes over time and segmented regression models of resistance outcomes incorporating the intervention (interruption at intervention plus 6 months). (DOCX)

S6 Table Sensitivity analysis 2: Results of ITSA of changes in antimicrobial resistance to targeted antimicrobials among community-associated coliform bacteraemia associated with the stewardship intervention (modelled interruption is date of primary care antimicrobial stewardship intervention plus 6 months) with 2005 data removed (including 2006 to 2015). ITSA, interrupted time series analysis.
(DOCX)

S7 Table Sensitivity analysis 2: Estimated absolute and relative changes, compared to levels predicted by prior trends, in resistance among community-associated coliform bacteraemia at 1.5 and 3.5 years after primary care antimicrobial stewardship intervention (modelled interruption is date of primary care antimicrobial stewardship intervention plus 6 months) with 2005 data removed (including 2006 to 2015). (DOCX)

## Abbreviations

AIC: Akaike Information Criterion
AMR: antimicrobial resistance
CHI: Community Health Index
CLSI: Clinical and Laboratory Standards Institute
EPOC: Effective Practice and Organisation of Care
HIC: Health Informatics Centre
ITSA: interrupted time series analysis
NHS: National Health Service
MIC: minimum inhibitory concentration
RECORD: REporting of studies Conducted using Observational Routinely collected health Data
SOP: Standard Operational Procedure
UBA: uncontrolled before–after
