# Supplementary material for: Changes in resistance among coliform bacteraemia associated with a primary care antimicrobial stewardship intervention: A population-based interrupted time series study
Source: PLoS Med. 2019 Jun 7;16(6):e1002825. doi: 10.1371/journal.pmed.1002825 (PMC6555503; doi:10.1371/journal.pmed.1002825)
Supplement: S1 Text — (DOC) [file pmed.1002825.s003.doc]

#### Data access

#### All datasets (“CHI”, community prescribing, microbiology and hospital admissions (SMR01)) were obtained through the Health Informatics Centre (HIC, [***https://www.dundee.ac.uk/hic/datalinkageservice/***](https://www.dundee.ac.uk/hic/datalinkageservice/)). HIC is a partnership between NHS Tayside, NHS Fife and the University of Dundee, which allows anonymised linkage of individual patient data from different electronic datasets using the Community Health Index (CHI) number, a unique patient identifier in use across all of NHS Scotland. HIC anonymizes the data transforming the CHI number into a unique anonymized patient identifier (the proCHI) which is consistent across all datasets within one project, enabling linkage at the individual level.

#### CHI dataset

#### The Community Health Index (CHI) dataset is a population register, which is used in Scotland for health care purposes. The CHI number identifies a unique person in the CHI register. CHI has information on date of birth (HIC anonymizes), gender, general practice the patient is registered with (anonymised practice code), and partial postcode which can be used to define small area characteristics applicable to individual patients.

#### Community prescribing

#### The community prescribing dataset includes information on all medications dispensed by a community pharmacist in NHS Tayside, including CHI of patient prescribed (proCHI provided to researchers), date dispensed, brand name, approved name, dose, quantity and route of administration. All prescription data were obtained from 2005 to 2015. Antimicrobials were identified based on approved name. Only oral antimicrobials were included and therefore topical, inhaled, and parenteral (injectable) antimicrobials were excluded.

#### Microbiology

#### All microbiology testing in Tayside generates an electronic record which HIC obtains directly from the laboratory data system. Microbiology data available include sample type and date of collection, microorganism isolated (or no growth), antimicrobial sensitivities, and source patient CHI (proCHI provided to researchers). Microbiology information is located across three different datasets, which can be linked by a unique sample identifier. A linked microbiology dataset was created with one row per sample for included results.

#### ‘MicroBiology_Tests’: type of test, e.g. urine culture, blood culture, wound swab, etc. Only blood cultures were included in this study.

#### ‘MicroBiology_Isolations’, which contains information about any specific microorganisms grown, weight of growth, and antimicrobial susceptibility. In this study, blood cultures with E. coli, Klebsiella spp. or Proteus spp.were included.

#### ‘MicroBiology_Lab’, which includes sample date and CHI (proCHI provided to researchers).

#### Scottish Morbidity Records 01 (SMR01)

#### The SMR01 dataset contains data for all acute hospital admissions for every patient, apart from psychiatric and maternity admissions, with date of admission and discharge, where the patient is admitted from and discharged to, and ICD-10 (International Classification of Diseases and Related Health Problems 10th version) codes for the medical conditions related to that admission.

#### The SMR01 dataset was used to define community-associated bacteraemias, by linkage with the microbiology dataset. Community-associated samples were defined as those taken on day 0, 1 or 2 of a hospital admission, from patients with no hospital admission in the previous 30 days (at least 30 days between discharge date from previous admission and sample date).
