# Supplementary material for: Changes in resistance among coliform bacteraemia associated with a primary care antimicrobial stewardship intervention: A population-based interrupted time series study
Source: PLoS Med. 2019 Jun 7;16(6):e1002825. doi: 10.1371/journal.pmed.1002825 (PMC6555503; doi:10.1371/journal.pmed.1002825)
Supplement: S1 Table — ITSA, interrupted time series analysis. (DOCX) [file pmed.1002825.s005.docx]

S1 Table. Interrupted time series analysis of rate of admission with community-associated coliform bacteraemia (modelled interruption is date of primary care antimicrobial stewardship intervention plus six months)

|  | Model estimates of baseline and trends | P value |
| --- | --- | --- |
| Baseline rate of admission with community-associated coliform bacteraemia (rate per 100,000 population)^a^ | 7.6 | < 0.001 |
| Baseline trend in rate (increase [+] or decrease [-] per quarter) ^b^ | 0.18 | 0.01 |
| Step-change at six months post-stewardship intervention (increase [+] or decrease [-] in rate per 1000 population)^b^ | 1.16 | 0.26 |
| Change in trend at six months post-stewardship intervention (increase [+] or decrease [-] in rate per quarter per 1000 population) ^b^ | -0.05 | 0.53 |

a. Rate of patients per 100,000 population admitted to hospital with community-associated coliform bacteraemia

b. Analysis pre-specified an expected delay of six months between the stewardship intervention at the start of quarter two 2009 and any change in resistance. The modelled interruption is therefore the start of quarter four 2009
