# Supplementary material for: Changes in resistance among coliform bacteraemia associated with a primary care antimicrobial stewardship intervention: A population-based interrupted time series study
Source: PLoS Med. 2019 Jun 7;16(6):e1002825. doi: 10.1371/journal.pmed.1002825 (PMC6555503; doi:10.1371/journal.pmed.1002825)
Supplement: S2 Table — (DOCX) [file pmed.1002825.s006.docx]

| Study year | Number of eligible bacteraemias | Number (%) resistant to each antimicrobial drug/class/combination | | | | |
| --- | --- | --- | --- | --- | --- | --- |
|  |  | Fluoroquinolones | Cephalosporins | Co-amoxiclav | Any two | All three |
| 2005 | 136 | 9 (6.6) | 8 (5.9) | 25 (18.4) | 9 (6.6) | 1 (0.7) |
| 2006 | 132 | 9 (6.8) | 9 (6.8) | 20 (15.1) | 5 (3.8) | 1 (0.8) |
| 2007 | 149 | 13 (8.7) | 12 (8.1) | 30 (20.1) | 10 (6.7) | 1 (0.7) |
| 2008 | 153 | 9 (5.9) | 31 (20.3) | 31 (20.3) | 10 (6.5) | 4 (2.6) |
| 2009 | 188 | 24 (12.8) | 17 (9.0) | 44 (23.4) | 24 (12.8) | 6 (3.2) |
| 2010 | 203 | 22 (10.8) | 26 (12.8) | 49 (24.1) | 22 (10.8) | 10 (4.9) |
| 2011 | 220 | 23 (10.4) | 17 (7.7) | 56 (25.4) | 21 (9.5) | 7 (3.2) |
| 2012 | 244 | 18 (7.4) | 30 (12.3) | 46 (18.8) | 18 (7.4) | 6 (2.5) |
| 2013 | 231 | 25 (10.8) | 30 (13.0) | 57 (24.7) | 26 (11.3) | 4 (1.7) |
| 2014 | 253 | 18 (7.1) | 33 (13.0) | 66 (26.1) | 29 (11.5) | 7 (2.8) |
| 2015 | 234 | 22 (9.4) | 31 (13.2) | 60 (25.6) | 26 (11.1) | 8 (3.4) |

S2 Table. Rates of resistance to individual antimicrobials and combinations of targeted antimicrobials among eligible community-associated bacteraemias per study year.
