# Supplementary material for: Changes in resistance among coliform bacteraemia associated with a primary care antimicrobial stewardship intervention: A population-based interrupted time series study
Source: PLoS Med. 2019 Jun 7;16(6):e1002825. doi: 10.1371/journal.pmed.1002825 (PMC6555503; doi:10.1371/journal.pmed.1002825)
Supplement: S3 Table — (DOCX) [file pmed.1002825.s007.docx]

| **Outcome** | **Model fit**  **Autocorrelation** | **Autocorrelation function (ACF) plot** | **Partial auto-correlation function (PACF) plot** | **Modelling strategy** |
| --- | --- | --- | --- | --- |
| Quinolone prescribing with no lagged terms | AIC 107.2  Durbin-Watson: 1.8 | 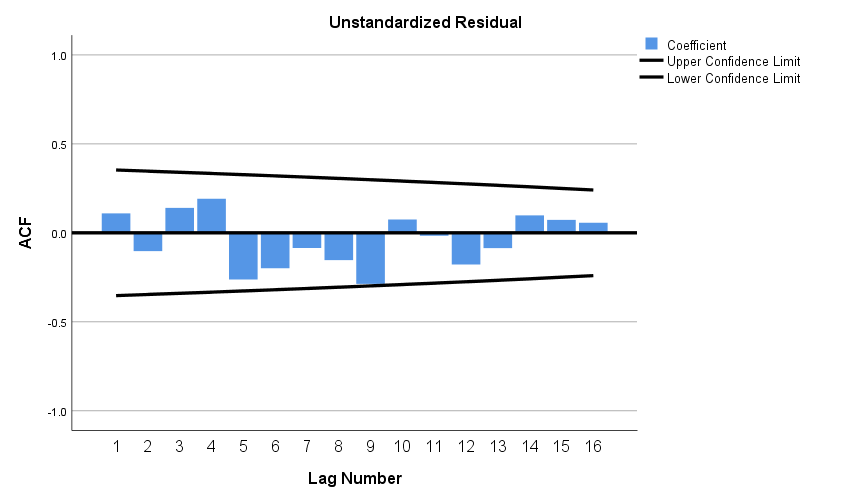 | 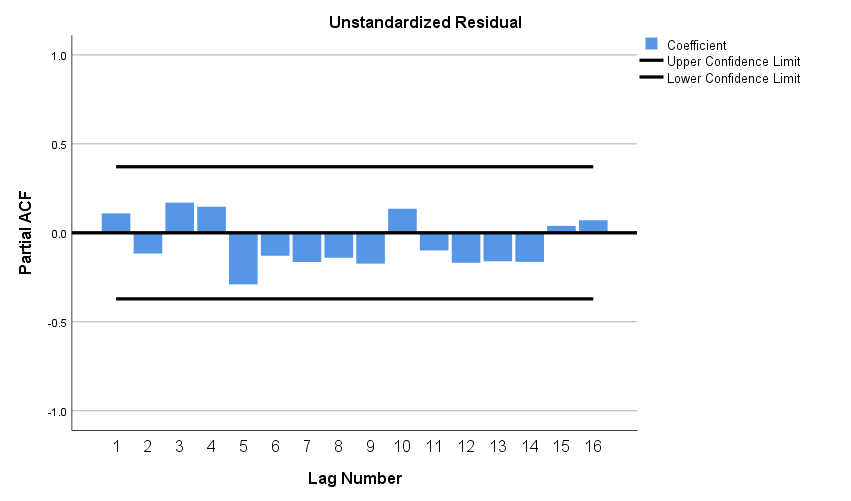 | No strong evidence of autocorrelation. |
| Cephalosporin prescribing with no lagged terms | AIC: 48.9  Durbin-Watson: 1.6 | 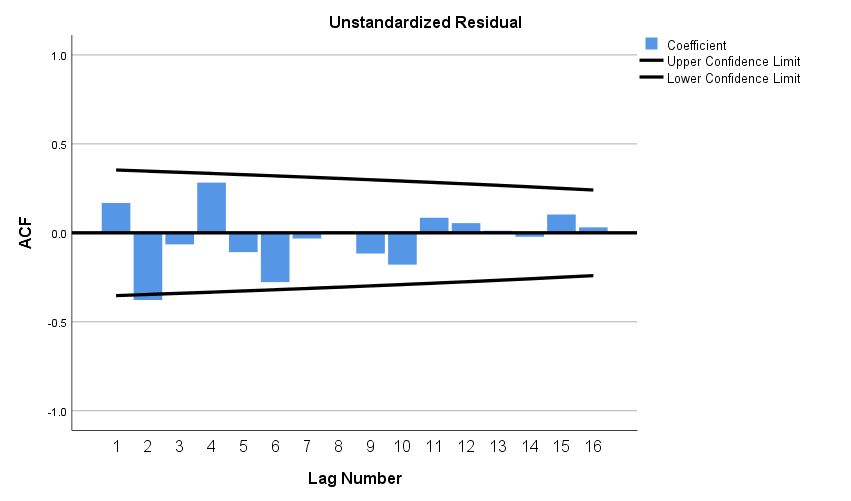 | 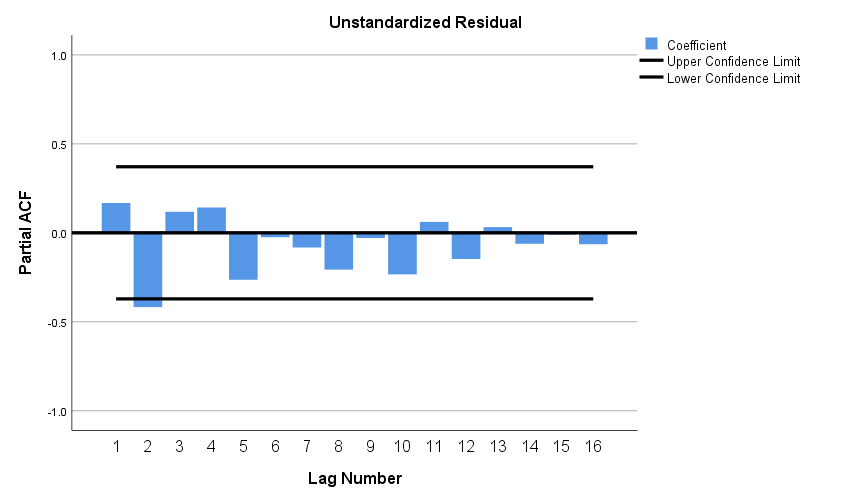 | There was marginal evidence of second order autocorrelation in the ACF and PACF plots, so fitting a lag 2 was explored. This did not improve model fit (AIC 48.7) or Durbin-Watson (1.3), and was not itself statistically significant in the model. The final model therefore does not include lagged terms. |
| Co-amoxiclav prescribing with no lagged terms | AIC: 61.4  Durbin-Watson: 1.8 | 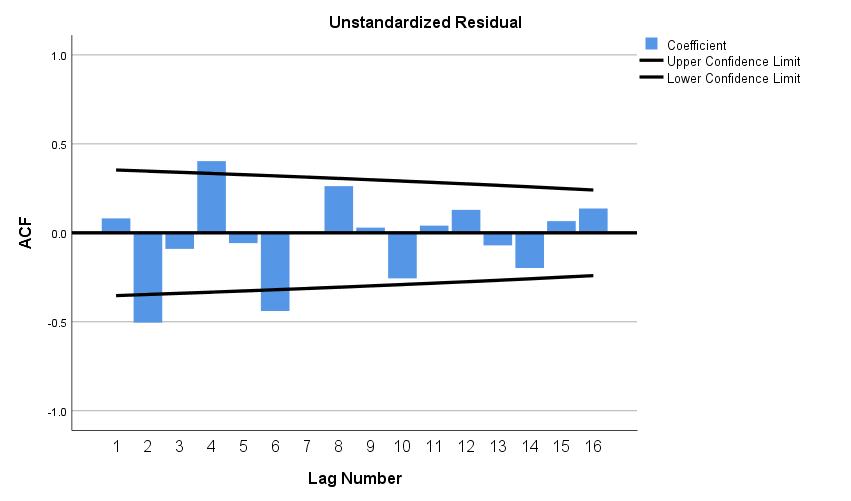 | 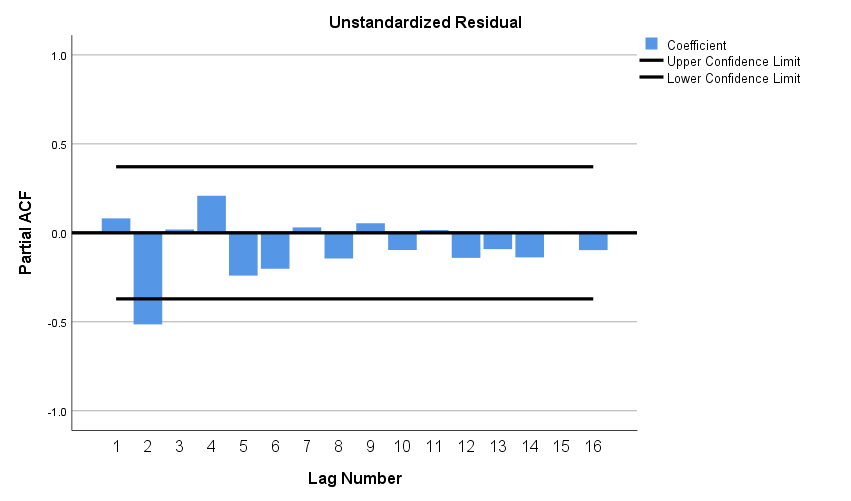 | The ACF and PACF plots show evidence of autocorrelation and/or seasonality. Fitting a lag 2 improved the model fit (AIC 50), but fitting a seasonal term (season defined as either ‘winter’ [quarters 1 and 4] or ‘summer’ [quarters 2 and 3] led to a better fitting model (AIC 42.9) and the season term itself was also statistically significant. The final model includes season. |
| Co-amoxiclav final model | AIC: 42.9  Durbin-Watson: 1.7 | 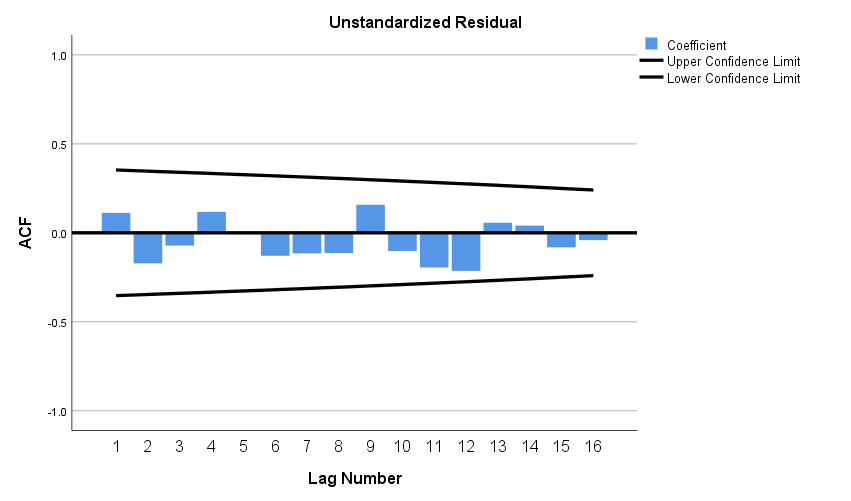 | 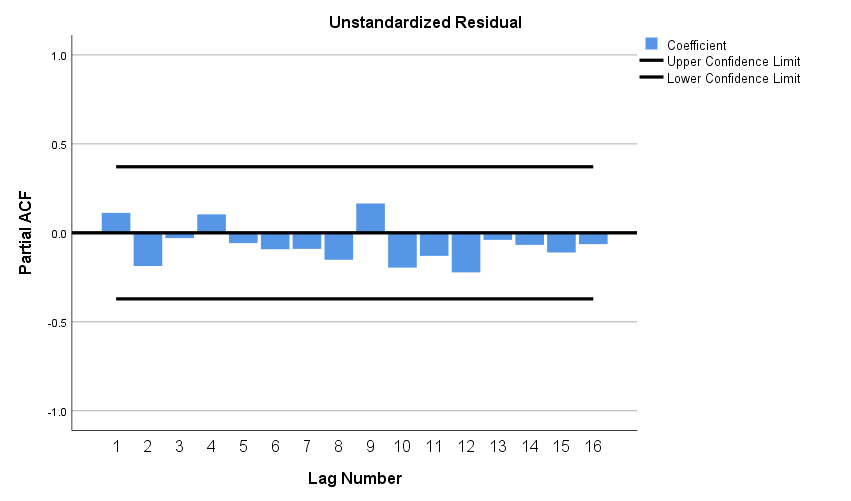 |  |

S3 Table. Assessment and accounting for autocorrelation in prescribing time series’ with final model selection.
