# Supplementary material for: Changes in resistance among coliform bacteraemia associated with a primary care antimicrobial stewardship intervention: A population-based interrupted time series study
Source: PLoS Med. 2019 Jun 7;16(6):e1002825. doi: 10.1371/journal.pmed.1002825 (PMC6555503; doi:10.1371/journal.pmed.1002825)
Supplement: S4 Table — (DOCX) [file pmed.1002825.s008.docx]

| **Outcome** | **Model fit**  **Autocorrelation** | **Autocorrelation function (ACF) plot** | **Partial auto-correlation function (PACF) plot** | **Modelling strategy** |
| --- | --- | --- | --- | --- |
| Quinolone resistance with no lagged terms | AIC: 444.2  Durbin-Watson: 2.2 | 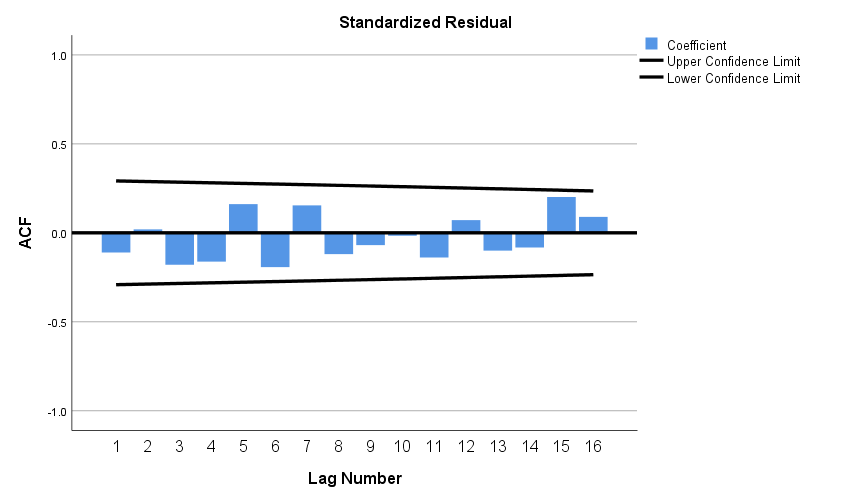 | 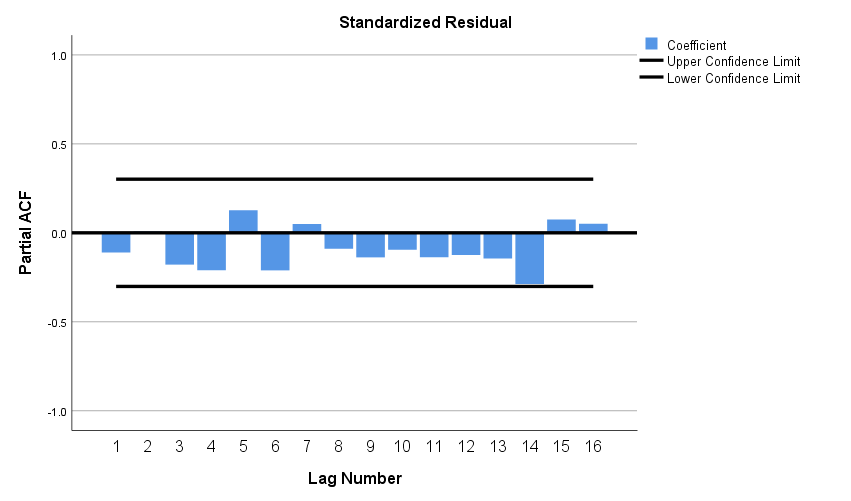 | No strong evidence of autocorrelation. |
| Cephalosporin resistance with no lagged terms | AIC: 481.4  Durbin-Watson 1.8 | 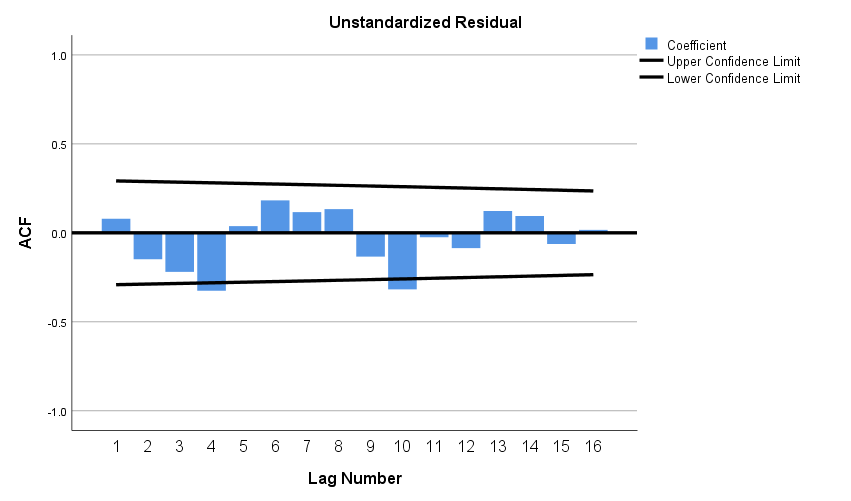 | 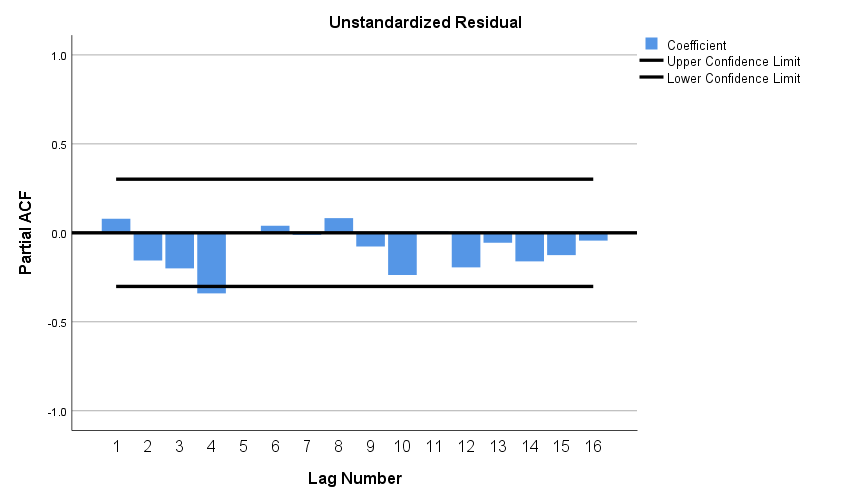 | ACF and PACF suggest presence of fourth order autocorrelation. A lag 4 was fitted, which improved both model fit and Durbin-Watson statistic. Therefore, the final model for cephalosporin resistance includes a lag 4 term. |
| Cephalosporin resistance final model | AIC: 478.8  Durbin-Watson: 1.91 | 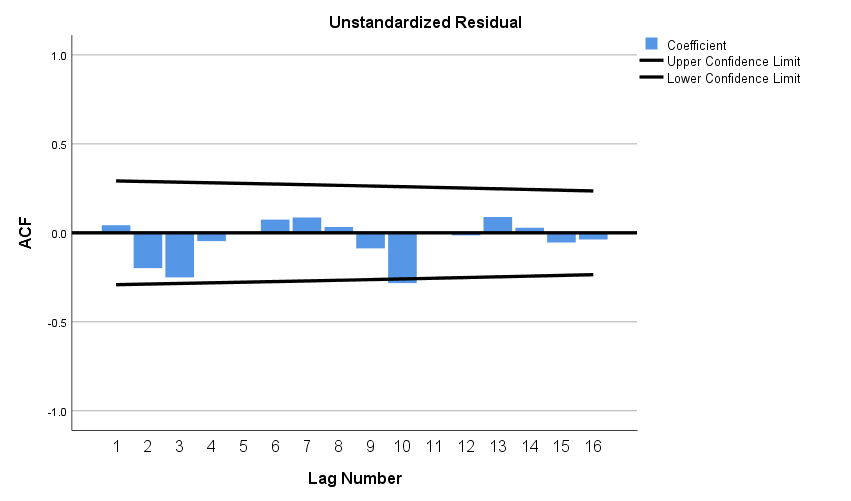 | 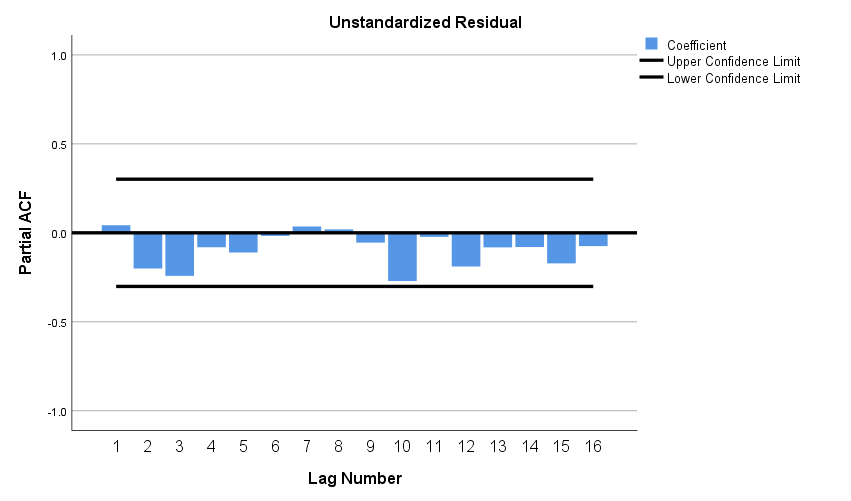 |  |
| Co-amoxiclav with no lagged terms | AIC: 507.1  Durbin-Watson: 2.5 | 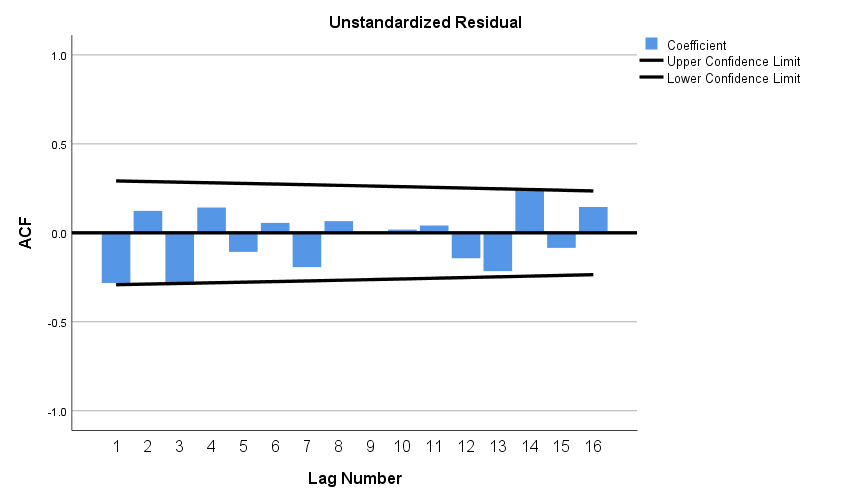 | 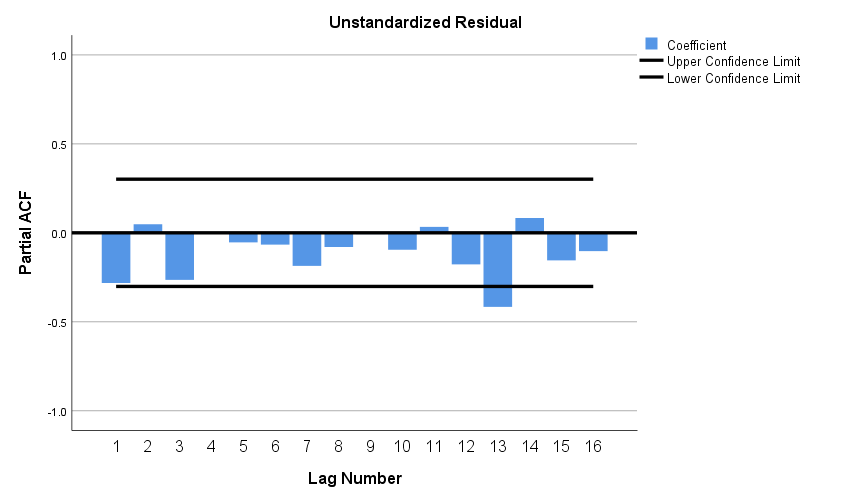 | There was marginal evidence of third order autocorrelation in the ACF plot, so fitting a lag 3 was explored, but this did not improve model fit (AIC 505.8) or the Durbin-Watson statistic (2.5) and was non-significant statistically in the model. Therefore, the final model does not include any lag terms. |

S4 Table. Assessment and accounting for autocorrelation in resistance time series’ with final model selection.
