# Supplementary material for: Changes in resistance among coliform bacteraemia associated with a primary care antimicrobial stewardship intervention: A population-based interrupted time series study
Source: PLoS Med. 2019 Jun 7;16(6):e1002825. doi: 10.1371/journal.pmed.1002825 (PMC6555503; doi:10.1371/journal.pmed.1002825)
Supplement: S5 Table — (DOCX) [file pmed.1002825.s009.docx]

| Outcome | Linear model AIC | Final segmented regression model AIC | Specification (any lags or seasonal variables included in the final segmented regression model) |
| --- | --- | --- | --- |
| Resistance (full time series 2005-2016)  Fluoroquinolones  Cephalosporins  Co-amoxiclav | 445.0  481.5  504.0 | 444.2  478.8  507.1 | No strong evidence of autocorrelation  Lag 4 included  No strong evidence of autocorrelation |
| Resistance (sensitivity analysis 2; 2006-2015)  Fluoroquinolones  Cephalosporins  Co-amoxiclav | 407.5  439.9  461.8 | 404.8  441.4  463.7 | No strong evidence of autocorrelation  No strong evidence of autocorrelation  No strong evidence of autocorrelation |

S5 Table. Sensitivity analysis 1: Comparing model fit for simple linear regression models of resistance outcomes over time and segmented regression models of resistance outcomes incorporating the intervention (interruption at intervention plus six months).
