# Supplementary material for: Changes in resistance among coliform bacteraemia associated with a primary care antimicrobial stewardship intervention: A population-based interrupted time series study
Source: PLoS Med. 2019 Jun 7;16(6):e1002825. doi: 10.1371/journal.pmed.1002825 (PMC6555503; doi:10.1371/journal.pmed.1002825)
Supplement: S6 Table — ITSA, interrupted time series analysis. (DOCX) [file pmed.1002825.s010.docx]

|  | Baseline resistance (rate per 1000 bacteraemias at start of time series)^a^ | Baseline trend (increase [+] or decrease [-] per quarter of rate per 1000 bacteraemias) | Step-change at six months post-intervention (increase [+] or decrease [-] in rate per 1000 bacteraemias) | Change in trend at six months post-intervention (increase [+] or decrease [-] in rate per quarter per 1000 bacteraemias) |
| --- | --- | --- | --- | --- |
| Fluoroquinolones  Cephalosporins  Co-amoxiclav | 25.4 (-24.9 to 75.7)  44.2 (-35.2 to 123.6)  106.5 (1.5 to 211.5) | +4.9 (1.0 to 8.9)  +5.7 (-0.5 to 11.9)  +7.9 (-0.4 to 16.1) | -12.9 (-55.2 to 29.4)  -42.5 (-109.3 to 29.4)  -30.6 (-119.0 to 57.8) | -5.9 (-10.3 to -1.6)  -4.8 (-11.7 to 2.0)  -6.6 (-15.7 to 2.5) |

S6 Table. Sensitivity analysis 2: Results of interrupted time series analysis of changes in antimicrobial resistance to targeted antimicrobials among community-associated coliform bacteraemia associated with the stewardship intervention (modelled interruption is date of primary care antimicrobial stewardship intervention plus six months) with 2005 data removed (including 2006 to 2015).
