# Supplementary material for: Changes in resistance among coliform bacteraemia associated with a primary care antimicrobial stewardship intervention: A population-based interrupted time series study
Source: PLoS Med. 2019 Jun 7;16(6):e1002825. doi: 10.1371/journal.pmed.1002825 (PMC6555503; doi:10.1371/journal.pmed.1002825)
Supplement: S7 Table — (DOCX) [file pmed.1002825.s011.docx]

|  | Absolute change at 1.5 years post-intervention compared to predicted (rate with resistant growth per 1000 coliform bacteraemias) | Absolute change at 3.5 years post-intervention compared to predicted (rate with resistant growth per 1000 coliform bacteraemias) | Relative change at 1.5 years post-intervention compared to predicted (rate with resistant growth per 1000 coliform bacteraemias) | Relative change at 3.5 years post-intervention compared to predicted (rate with resistant growth per 1000 coliform bacteraemias) |
| --- | --- | --- | --- | --- |
| Fluoroquinolones  Cephalosporins  Co-amoxiclav | -36.6 (-90.6 to 17.2)  -61.9 (-147.1 to 23.3)  -56.9 (-169.5 to 55.7) | -84.2 (-166.4 to -2.0)  -100.7 (-230.5 to 29.2)  -109.7 (-281.4 to 61.5) | -25.4% (-54.5 to 3.7)  -34.2% (-67.5 to -0.9)  -19.3% (-169.6 to 55.7) | -45.7% (-69.8 to -21.7)  -44.4% (-76.1 to -12.7)  -30.6% (-63.7 to 2.4) |

S7 Table. Sensitivity analysis 2: Estimated absolute and relative changes, compared to levels predicted by prior trends, in resistance among community-associated coliform bacteraemia at 1.5 and 3.5 years after primary care antimicrobial stewardship intervention (modelled interruption is date of primary care antimicrobial stewardship intervention plus six months) with 2005 data removed (including 2006 to 2015).
